# The Influence of the Deformation Method on the Microstructure and Properties of Magnesium Alloy Mg-Y-RE-Zr

**DOI:** 10.3390/ma15062017

**Published:** 2022-03-09

**Authors:** Iwona Bednarczyk, Dariusz Kuc

**Affiliations:** Faculty of Materials Engineering, Silesian University of Technology, 44-100 Gliwice, Poland; dariusz.kuc@polsl.pl

**Keywords:** magnesium alloys, severe plastic deformation, microstructure, STEM/TEM, grain refinement, mechanical properties

## Abstract

This article presents the influence of the applied extrusion method on the microstructure and mechanical properties of the WE43 magnesium alloy. The materials for tests were ingots made from magnesium alloy, with dimensions of 40 × 90 mm, marked with the symbol WE43. Two extrusion methods were used: the classic one—concurrent extrusion, and the complex one—concurrent extrusion with a reversible die (KoBo). As a result of the application of deformation processes, rods were obtained. The implemented deformation methods made it possible to determine the influence of the deformation process parameters on changes in the structure and properties of the WE43 alloy. In addition, compression tests were performed to determine the values of the yield stress and to analyze changes in the microstructure after plastic deformation. The hot plastic deformation activation energy and the process parameters, for which the course of plastic flow is affected by the presence of twins in the microstructure, were determined for the WE43 alloy. The effects of superplastic flow at 350 °C (250% elongation) and microstructure refinement (d = 1 µm) were demonstrated after applying the KoBo method. The results will be useful in the development of forming technology of selected construction elements, which serve as light substitutes for currently used materials.

## 1. Introduction

Currently, the interest in magnesium alloys in the aviation and automotive industries is growing. The production of semi-products and products from magnesium alloys is mainly based on the technology of casting and the good casting properties of magnesium alloys. Magnesium alloys are characterized by a higher specific strength (ratio of strength to density) than that of the alloys of other metals [1,2,3]. Their disadvantage, however, is their low plasticity, which limits the possibility of producing structural elements from this group of alloys using plastic working methods. Therefore, new methods of processing this group of materials are sought, and among them is the use of plastic working in complex deformation states. In recent years, there has been an increasing interest of the industry in light alloys, mainly magnesium alloys, and the number of scientific publications in the field of research on magnesium alloys has also steadily increased. Current research into magnesium alloys is focused on developing new alloys; improving casting technology, as well as plastic processing; and increasing the ductility of these alloys [4,5,6]. The few indications in the literature also suggest that there are opportunities to improve the strength properties of magnesium alloys by using non-conventional deformation methods, such as SPD. Severe plastic deformation (SPD) techniques have proven to be effective processes that can be used to achieve significant grain refinement in metallic materials down to the sub-micrometer range. However, processing magnesium alloys with SPD is not trivial due to the limited ductility of these alloys. The grain refinement produced by SPD leads to significant changes in the mechanical properties of magnesium alloys. The ultrafine grains are stable at high temperatures, and superplasticity is observed. The application of unconventional methods of deformation for magnesium alloys results in the alloys’ increased plasticity. This is why new trends are observed in the modification of classic plastic forming methods, which aim to obtain new products with improved qualities [7,8,9]. There are tests being conducted at the Department of Advanced Materials and Technologies of the Silesian University of Technology as part of research tasks, including tests of plastic forming of the WE43 alloy with the use of conventional methods (direct extrusion) and extrusion with the KoBo method. The latter belongs to the category of unconventional methods of plastic forming with the use of large deformations, called SPD—severe plastic deformation. In KoBo extrusion, there is an additional oscillatory movement of the die in the orthogonal plane in the direction of extrusion (flow of material). Its application allows for the replacement of high-temperature deformation, with the process being conducted at cold temperatures and at a high speed, without initial heating of the charge, and with a much increased degree of deformation with a lower deformation work value. An additional advantage of this process is the achievement of beneficial mechanical properties, which are not typically observed in the case of other deformation methods [10,11,12,13,14,15].

This paper presents the results of a study on the changes in the structure and properties of the WE43 alloy after deformation using the KoBo method. On the basis of tensile tests, the plasticity changes were determined at temperatures ranging from 250 °C to 350 °C. Compression tests conducted at temperatures ranging from 250 °C to 350 °C and at a variety of strain rates (0.01, 0.1, and to 1 s^−1^) provided important data concerning the influence of process parameters on flow stress and microstructural changes in relation to the recrystallization process. The dependencies of flow stress and deformation on the Zener–Hollomon parameter were determined. After the deformation process using the KoBo method and classical extrusion, the mechanical properties of the WE43 alloy were evaluated. Static tensile tests were performed at temperatures of 300 °C and 350 °C and at strain rates of 0.001 and 0.0001 m·s^−1^.

## 2. Materials and Methods

The test material consisted of ingots made from the WE43 magnesium alloy with the dimensions 120 × 65 mm. Chemical compositions of the tested alloys are presented in Table 1. The test material was a continuously cast commercial electron magnesium alloy WE43. After casting, the alloy was subjected to solution heat treatment at a temperature of 525 °C with a soaking time of 8 h, and it was then air cooled. The presence of neodymium and yttrium in the chemical composition of the WE43 alloy positively influences the improvement in mechanical properties at both room and elevated temperatures.

**Figure 1:**
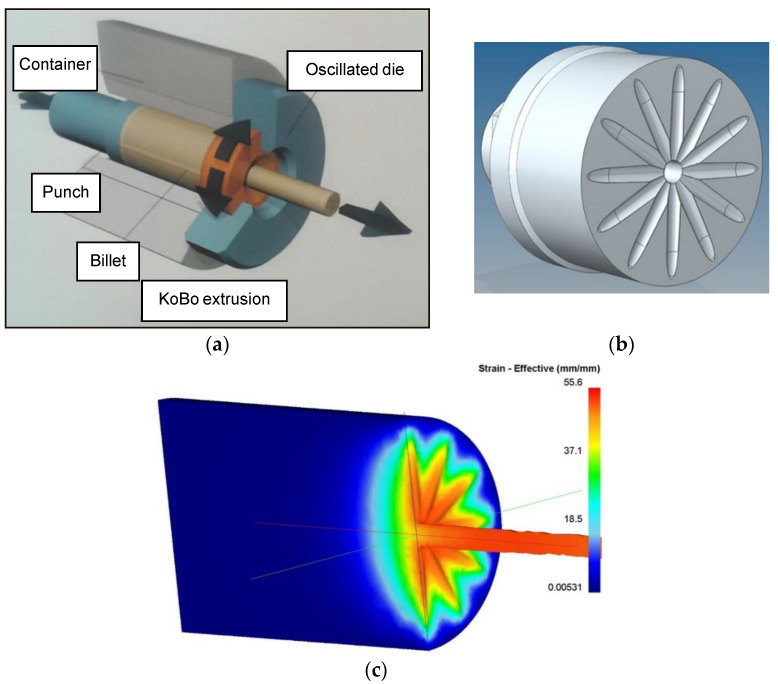
Scheme of KoBo extrusion process (**a**), view of die used for extrusion (**b**), result of FEM computer simulation of extrusion process, (**c**) deformation intensity distribution.

The ingots underwent the process of extrusion to a diameter of 40 mm. Next, they were subject to annealing for 40 min at a temperature of 400 °C and were then air cooled. Severe plastic deformation tests were carried out with a modernized KoBo horizontal hydraulic press. The WE43 alloy was directly extruded using the KoBo method at room temperature without prior heating, with a shift speed of inverted die of 0.33 mm/s, a die torsion angle of ±8°, and a frequency of 5 Hz. Rods with diameters of 8 and 6 mm were obtained as a result of extrusion. The processing degree, λ, calculated for them equaled 100. The material was intensively cooled with water at the press throat outlet (Figure 1).

In addition, for comparison, a conventional extrusion process was carried out at 400 °C on a Hydromet press (Hydromet, Bytom, Poland) in which rods with a diameter of 8 mm were obtained. Cylindrical samples, 10 mm in diameter and 12 mm in height, were used to obtain the necessary data from the hot deformation simulator, Gleeble 3800 (Dynamic Systems Inc., New York, NY, USA). They were subjected to uniaxial compression tests. The tests associated with the study of dynamic softening were conducted at T = 250, 300, and 350 °C and at nominal strain rates of ε = 0.01, 0.1, and 1 s^−1^. After attaining the equivalent strain ε = 1, the samples were cooled in water. From the values of peak stress σ_pp_ of particular stress–strain curves, the value of the activation energy in hot forming was determined, and, then, by means of this value, the determination of the kinetics of dynamic recrystallization followed (specifically, the strain to reach the peak, ε_p_, corresponding to the dynamic recrystallization start) as a function of the Zener–Hollomon parameter (Z).

The apparent activation energy value Q (J·mol^−1^) in hot forming is a key material constant needed, e.g., for the calculation of the temperature compensated strain rate represented by the Zener–Hollomon parameter Z(s^−1^):

(1) $$Ζ=\dot{\varepsilon }\exp\left[\frac{Q}{\mathrm{RT}}\right],$$

Knowledge of the given material enables one to effectively predict the maximum flow stress value σ_pp_ (MPa) or the strain value ε_p_[-] corresponding to the start of the dynamic recrystallization at the given temperature T(K) and strain rate $\dot{\varepsilon }$(s^−1^). The Q-value should ideally be the material constant depending only on the chemical composition and structure of the given material. The hyperbolic law in the Arrhenius-type equation is conventionally used for its determination [7]:

(2) $$\dot{\varepsilon }=C\cdot \exp\left(\frac{-Q}{R\cdot T}\right)\cdot {\left[\sin h\left(\alpha \cdot \sigma_{\max}\right)\right]}^{n},$$

where C (s^−1^), n [-], and α(MPa^−1^) are other material constants; R = 8.314 J·mol^−1^·K^−1^; and σ_pp_ (MPa) is the flow stress corresponding to the peak stress on stress–strain curve.

This relationship is solved using a simple graphic method based on the repeated application of linear regression. A particularity of the hyperbolic function is used in this calculation, which enables the simplification of Equation (2) for low stress values (i.e., for a high-temperature level) to the form of the Arrhenius power law:

(3) $$\dot{\varepsilon }=C_{1}\exp\left[\frac{-Q}{R.T}\right]\sigma_{\max}^{n},$$

and, vice versa, for high stress values (i.e., for a low-temperature level), to the form of the exponential law:

(4) $$\dot{\varepsilon }=C_{2}\exp\left[\frac{-Q}{\mathrm{RT}}\right]\exp\left({\beta \sigma }_{\max}\right),$$

where C_1_ (s^−1^), C_2_ (s^−1^), and β (MPa^−1^) are the material constants. The constant α in Equation (2) is given by the following relation:

(5) $$\alpha =\frac{\beta }{n},$$

For the selected level at a high temperature, the constant n is determined by linear regression of the experimental data in coordinates $\ln\sigma_{\max}\sim \ln\dot{\varepsilon }$, and for the selected level at a high temperature, the constant β is obtained by linear regression in coordinates $\sigma_{\max}\sim \ln\dot{\varepsilon }$. Once the quantity α has been calculated in accordance with Equation (5) the constants Q and C in the equation can be calculated by a final linear regression of all the data plotted in the coordinates $T^{-1}=\left(\ln\dot{\varepsilon }-n\cdot \sin h(\propto \cdot \sigma_{\max}\right)$.

Such estimates of constants n and β can be strongly influenced by the choice of the appropriate temperature level, as well as by the dispersion of the experimental data. This defect was eliminated by using the specially developed ENERGY 4.0 [16] interactive software. The program uses the values of n and β determined in the previous procedure only as a first estimate of the parameters for the final refinement of the multiple nonlinear regression of all data corresponding to the equation. The load ε_p_[-] corresponding to the peak strain can be described, depending on parameter Z, by dependence:

(6) $$\varepsilon_{p}=U\cdot Z^{W},$$

The value of the strain rate sensitivity coefficient (m) can be evaluated as the slope of lnσ − ln $\dot{\varepsilon }$, which is defined as

(7) $$m=\frac{\partial {\ln\sigma }_{p}}{\partial \ln\dot{\varepsilon }}\left|\varepsilon_{p},T\right|$$

where σ_p_ is the maximum flow stress (MPa), $\dot{\varepsilon }$ is the strain rate (s^−1^), and T is the deformation temperature (°C).

**Figure 2:**
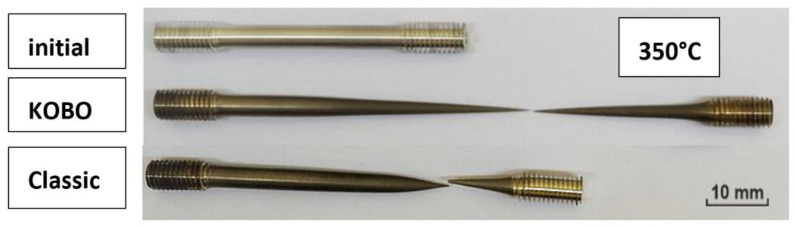
The appearance of samples for the tensile test and after the test.

Examination of the microstructure of the WE43 alloy was performed on the cross-section parallel to the axis of the sample. The samples were included in a conducting material and etched in a solution intended for etching magnesium alloys, containing 4.2 g (NO_2_)3C_6_H_2_OH (picric acid), 70 mL C_2_H_5_OH (ethyl alcohol), 10 mL H_2_O (water), and 10 mL CH_3_COOH (glacial acetic acid). The microstructure of the WE43 alloy was analyzed in the initial state after deformation using an Olympus GX71 light microscope (Olympus, Glasgow, UK) in bright field mode. Additionally to the analysis of the microstructure, quantitative and qualitative analyses were conducted with the use of the Metilo program [17]. The grain size was measured using a surface method based on images recorded on a light microscope. The studies of the microstructure were supplemented by observation using a scanning transmission electron microscope (STEM) (Hitachi HD-2300 A, Hitachi Science&Technology, Tokyo, Japan) equipped with a field emission gun (FEG) operated at 200 kV for microstructure characterization on longitudinal sections of the extruded billet. For microstructure examination, we used transmitted electron (TE) imaging. For STEM investigations, foils with a diameter of 3.0 mm after electrolytic thinning were used. (Figure 2). Tensile tests were carried out on a ZWICK testing machine (Zwick Roell AG, Ulm, Germany) with a maximum force of 250 kN. Results were recorded on a computer using TestXpert 2 software (AG, Ulm, Germany). The tests were performed at ambient temperature (RT), 300, and 350 °C with a constant strain rate of 1 × 10^-4^ s^−1^ (Figure 2).

## 3. Results

**Figure 3:**
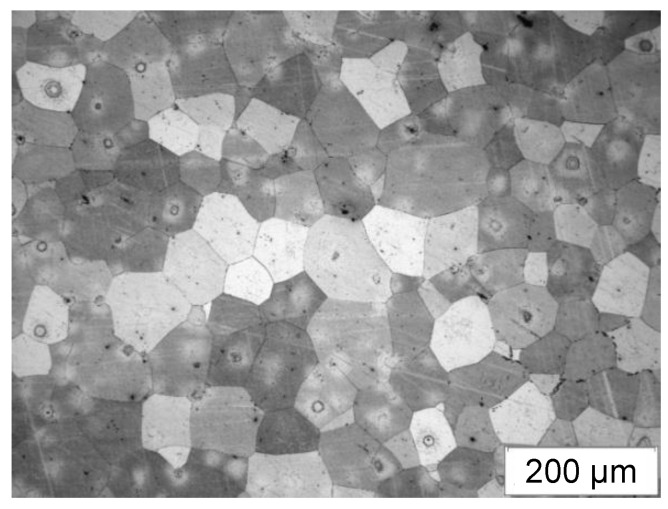
Microstructure of WE43 alloy after casting process and solution heat treatment.

Figure 3 presents an example microstructure of the WE43 alloy after the processes of casting and heat treatment. After the casting process and heat treatment, the WE43 alloy was characterized by a coarse-grained microstructure with varied sizes of grain (Figure 3). In the initial state, the grain mean diameter was about 70 μm (Table 2). The phase composition of the WE43 alloy in its initial state was identified using an X-ray phase analysis. The presence of α-Mg solid solution and phase Mg_41_Nd_5_ was demonstrated in previous studies [18].

**Figure 4:**
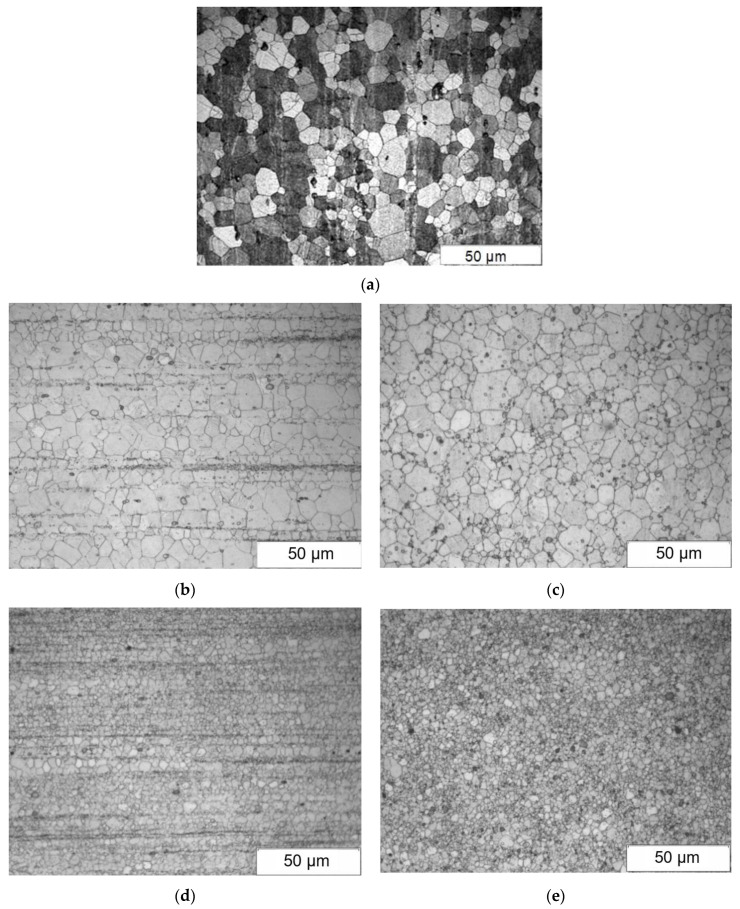
Microstructure of WE43 alloy after plastic deformation: (**a**) direct extrusion; (**b**,**c**) KoBo deformation, rod with diameter of 8 mm; (**d**,**e**) KoBo method extrusion, rod with diameter of 6 mm.

Figure 4 presents example microstructures of the WE43 alloy after concurrent extrusion (Figure 4a) and extrusion using the KoBo method (Figure 4b,c).

The analysis of the microstructure after extrusion with the KoBo method showed the presence of a recrystallized structure. The analysis of the microstructure of the WE43 alloy after extrusion and KoBo extrusion revealed significant grain refinement. Precipitations observed in the microstructure of the WE43 alloy are located mainly on grain boundaries (Figure 4b) and form a band layout (Figure 4c). By comparison of the achieved microstructure after the KoBo process with the microstructure after classic extrusion of the WE43 alloy, it can be concluded that there is significant fineness of structure present after applying the KoBo method. Grain with an average diameter of about 55 μm (rod with diameter of 8 mm) and 1µm (rod with diameter of 6 mm) was obtained, which was smaller than the grain obtained after classic extrusion, where the average grain diameter was 68 μm. (Figure 4, Table 2).The analysis of the microstructure of the rods after extrusion and extrusion using the KoBo method showed that the microstructure of the WE43 alloy was refined due to the recrystallization process (Figure 4). An average grain size below 55 µm was obtained. The highest grain refinement (1 µm) was obtained for a rod with a diameter of 6 mm (Table 2). The analysis of the microstructure of the WE43 alloy after deformation using the KoBo method was supplemented with a quantitative analysis. The results of the quantitative characterization of WE43 are presented in Table 2.

Samples after Kobo extrusion are characterized by lower inhomogeneity of the microstructure than that achieved when using conventional (direct) extrusion, as evidenced by the lower value of the variation coefficient S(A) (Table 2). Samples extruded to a smaller diameter are characterized by a homogeneous fine-grained microstructure.

**Figure 5:**
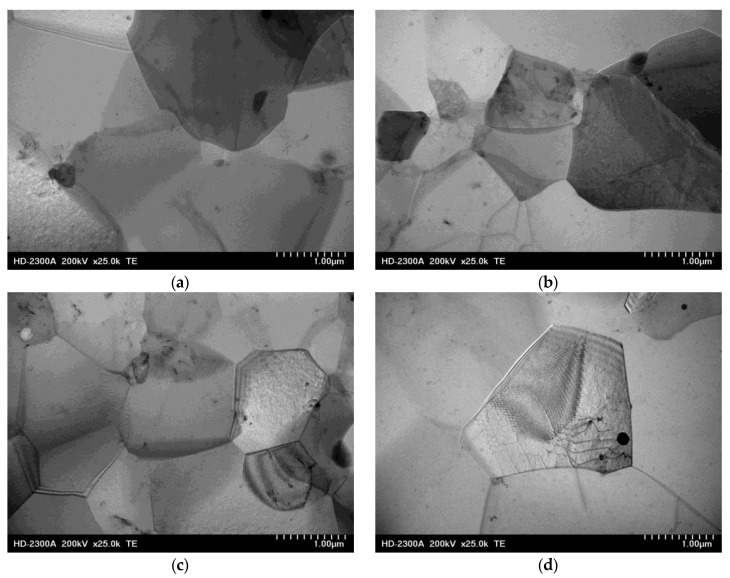

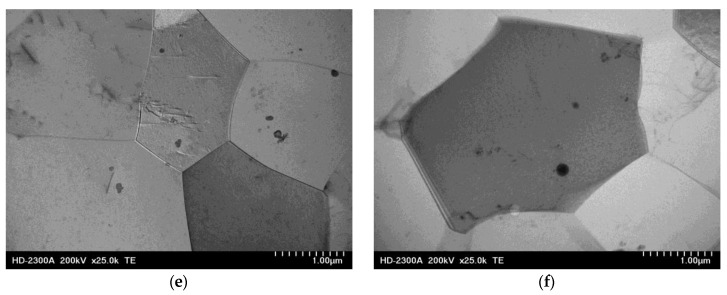
TEM images showing the characteristic microstructures of WE43 alloy after deformation with KoBo method, transmitted electron (TE) images. (**a**–**c**) Grain/subgrain; (**d**) migration of a wide-angle grain boundary; (**e**,**f**) recrystallized areas, grains free of dislocations.

Figure 5 presents an example microstructure of the WE43 alloy after the deformation process with the use of scanning transmission microscopy. Investigations of the substructure after the KoBo extrusion process revealed the recrystallization process as the mechanism of structure restoration (Figure 5). Grains/subgrains with a low dislocation density and recrystallization nuclei were revealed (Figure 5a–c). It was found that subgrain boundaries lost the nature of dislocation pile-ups and turned into wide-angle boundaries (Figure 5d). It was also observed that the recrystallization process induced the formation of equiaxial dislocation-free grains (Figure 5e,f). In addition, the presence of precipitates (Figure 5d–f) was found on grain/subgrain boundaries in the microstructure of the WE43 alloy.

**Figure 6:**
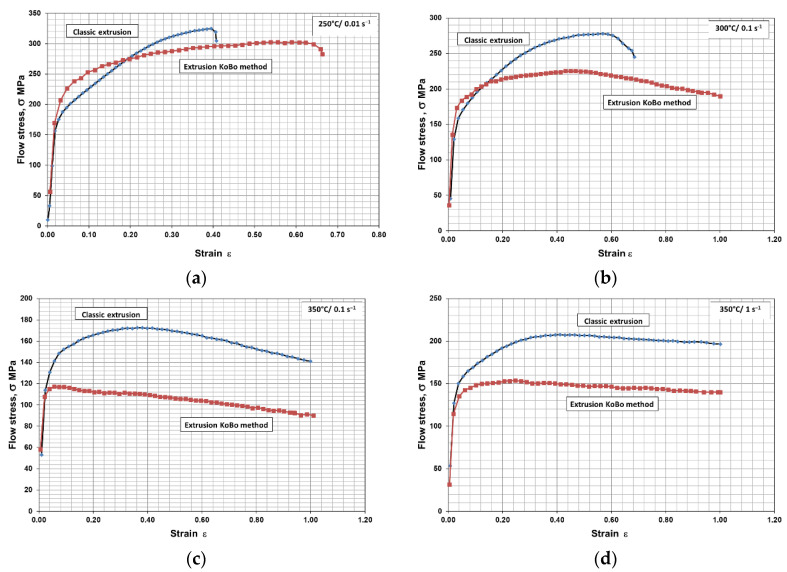

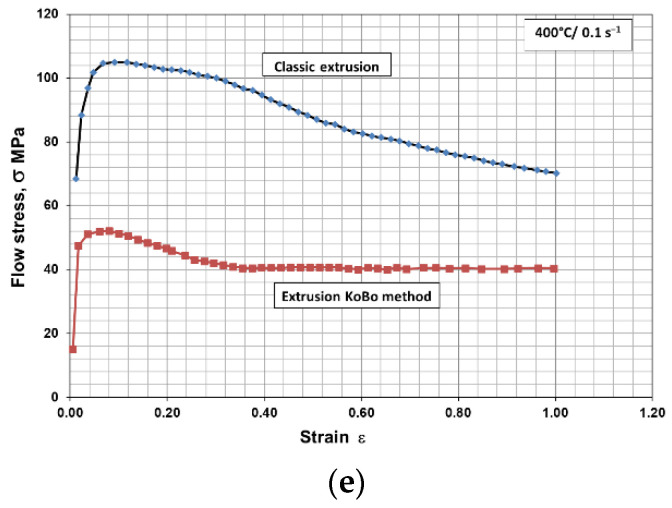
Flow stress of the investigated WE43 alloy after deformation at various temperatures: (**a**) 250 °C—0.01 s^−1^, (**b**) 300 °C—0.1 s^−1^, (**c**) 350 °C—0.1 s^−1^, (**d**) 350 °C—1 s^−1^, (**e**) 400 °C—0.1 s^−1^.

Example flow curves that show the influence of temperature on the flow stress of the tested WE43 alloy are presented in Figure 6. A curve that initially had a concave shape, which is connected to the intensive course of twinning in the microstructure, was achieved. After reaching the peak stress, together with the increase in deformation, the stress intensity dropped. An increase in strain rate also favored the achievement of a characteristic course of flow stress changes during deformation, within which twinning took place in the microstructure. Deformation at temperatures of 250 °C and 300 °C caused the sample to crack at strains of 0.4 and 0.6, and the predefined strain of ε = 1 was not achieved (Figure 6a,b).

From the flow curves, mathematical relationships were developed between the process parameters and the mechanical properties determined in a plastometric compression test. From the stress–strain curves (Figure 6), the values of maximum flow stress σ_pmax_ and the corresponding strain ε_p_ were read out. A comparison of the stress–strain curves for the WE43 alloy specimens after coextrusion and combined extrusion after compression at 400 °C and at a rate of 0.1 s^-1^ is shown in Figure 5e. The compressed specimens strengthen, and when the maximum stress is reached, this value is reduced to a preset strain of ε = 1. The value of stress is much higher for the sample after coextrusion than after combined extrusion. In the compressed sample, extruded using the KoBo method, after strengthening and exceeding a strain of 0.3, the stress remains at a constant value, which shows that the so-called “steady state flow” has been reached. This testifies to a complete and cyclic dynamic recrystallization. The determined values of flow stress σ_pmax_ and the corresponding strain ε_p_ for samples—which, after extrusion and after extrusion using the KoBo method, were subject to a compression test—are presented in Table 3. In the whole range of the analyzed temperature and the given strain rate, for the samples, after deformation using the KoBo method, the obtained values of the maximum flow stress σ_pmax_ were lower than the values obtained after classic extrusion (Table 3, Figure 6).

From the values of peak stress σ_pmax_ of particular stress–strain curves, the value of the activation energy in hot forming was determined, and other constants in Equations (2) and (6) were calculated by applying the procedure described above. The effect of deformation heat generation was taken into account, and only real and not nominal forming temperatures were considered. Similarly, the mean strain rates were calculated for the relevant peak-stress values because these values can significantly differ from the nominal value during the initial phase of a compression test. The calculated constants in Equations (2) and (6) are summarized in Table 4.

**Figure 7:**
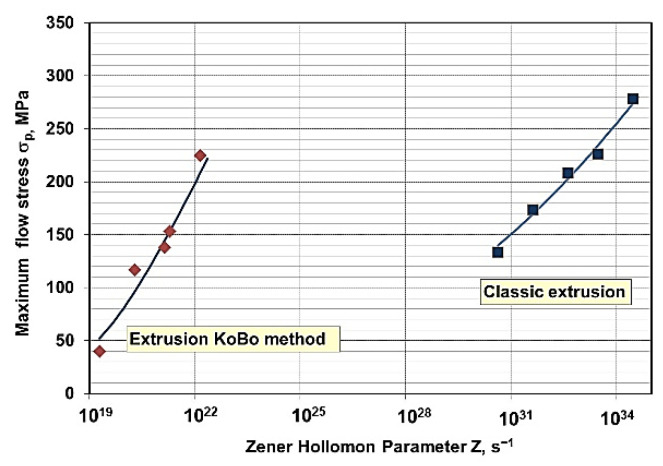
Comparison of the maximum flow stress σ_pmax_—Zener–Hollomon parameter Z dependence for WE43 alloy after concurrent and combined extrusion; markers—experimental data, solid line of calculation.

On the basis of the determined material constants, it can be stated that the dependence:

(8) $$\sigma_{\mathrm{pmax}}=\frac{1}{\alpha }a\sin h\left({\left(\frac{Z}{C}\right)}^{n}\right),$$

makes it possible to calculate the flow stress σ_pmax_ as a function of temperature and strain rate described with parameter Z for both samples extruded in the conventional way and those extruded concurrently. These data are presented in the graph (Figure 7). The differences in the Z parameter values for the two variants analyzed are due to the significantly lower activation energy Q for the alloy samples extruded using the KoBo method compared to the conventionally deformed alloy, i.e., 389.2 kJ/mol and 253.9 kJ/mol, respectively (Table 4). The ε_p_ (Z) dependence was not determined, since a low correlation was observed between the experimental data and those calculated according to the exponential relation.

**Figure 8:**
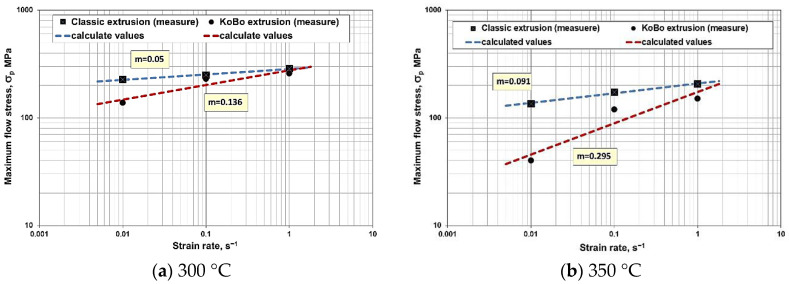
The strain rate sensitivity coefficient (m) depending on strain rate in the logarithmic system (**a**) 300 °C, (**b**) 350 °C.

The m-factor was calculated for strain temperatures of 300 and 350 °C. The values of σ_p_ as a function of $\dot{\varepsilon }$ presented in logarithmic order show high consistency in the form of a linear function. For a strain temperature of 300 °C, the values of the m-factor are low: 0.05 and 0.136 for conventional extrusion and KoBo extrusion, respectively (Figure 8a). Higher strain rate sensitivity is observed at 350 °C. Conventionally extruded samples show low strain rate sensitivity, while the opposite is true for the KoBo extruded samples, which are characterized by grain fineness. The result obtained for KoBo extruded samples (m = 0.295) indicates increased ductility. It can be concluded that the material becomes superplastic under these conditions (Figure 8b).

**Figure 9:**
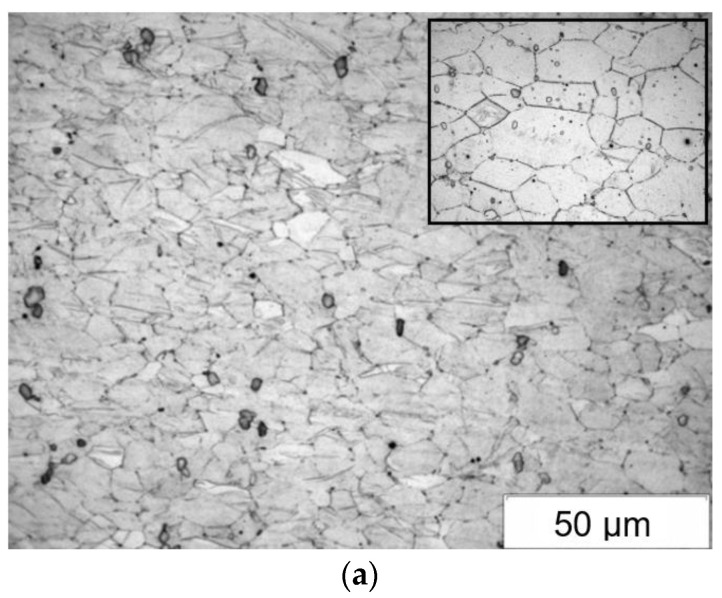

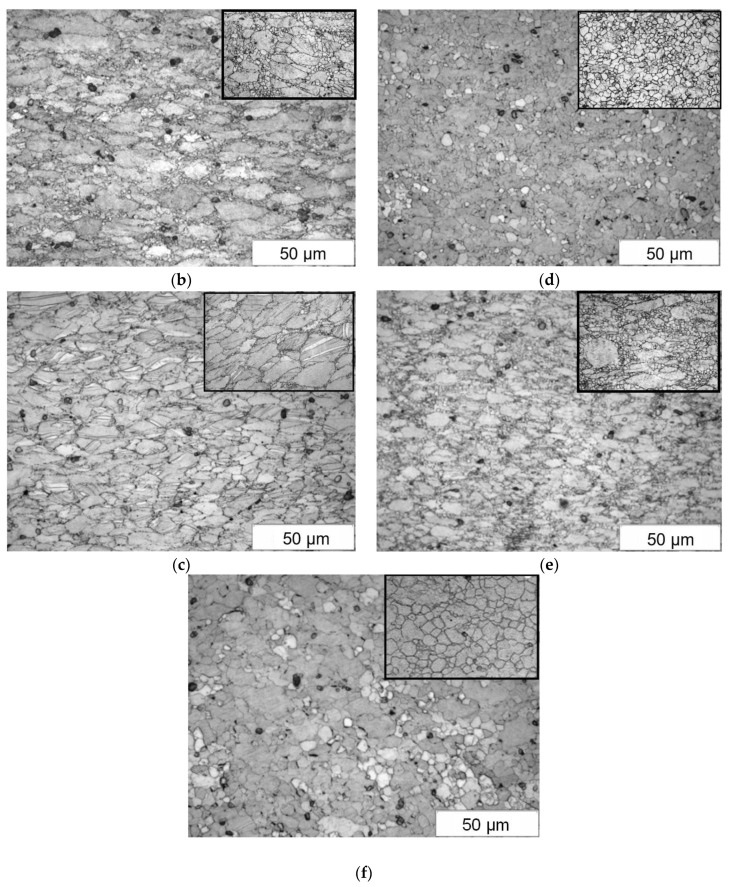
The microstructure of the investigated WE43 alloy after compression at a temperature of 250–400 °C and at a strain rate of 0.01–0.1 s^−1^. (**a**) 250 °C—0.01 s^−1^; (**b**) 300 °C—0.01 s^−1^; (**c**) 300 °C—0.1 s^−1^; (**d**) 350 °C—0.01 s^−1^; (**e**) 350 °C—0.1 s^−1^; (**f**) 400 °C—0.1 s^−1^.

Figure 9 presents the WE43 alloy microstructures after an axial-symmetric compression test in the temperature range of 250–400 °C and at a strain rate of 0.01–1 s^−1^. The WE43 microstructure at 250 °C and at a strain rate of 0.01 s^−1^ was observed to be elongated in the direction of strain, and in the grains within this strain, deformation twins were present (Figure 9a). A further increase in temperature, i.e., 300 °C, revealed in the microstructure of the WE43 alloy elongated primary grains and new, fine equiaxial dynamically recrystallized grains within the boundaries of the primary grain. This testifies to an incomplete recrystallization process (Figure 9b). An increase in the strain rate of 0.1 s^−1^ at 300 °C caused multiple deformation twins to appear within the primary grains (Figure 9c). Increasing the deformation temperature to 350 °C led to an intensification of the recrystallization process, especially after compression at a low speed of 0.01 s^−1^. In the microstructure after compression at 0.01 s^−1^, full recrystallization was found to occur, which was manifested by fine grains and a lack of elongated primary grains (Figure 9d). Increasing the strain rate to 0.1 s^−1^ made it impossible for full recrystallization to take place—the microstructure was bi-modal and consisted of elongated primary grains and fine recrystallized grains (Figure 9e). A further increase in the strain rate to 1 s^−1^ led to a similar type of microstructure, but the recrystallized grains were much smaller. Further increasing the deformation temperature to 400 °C led to a microstructure composed exclusively of recrystallized grains whose average size was larger than after compression at a temperature of 350 °C (Figure 9f).

**Figure 10:**
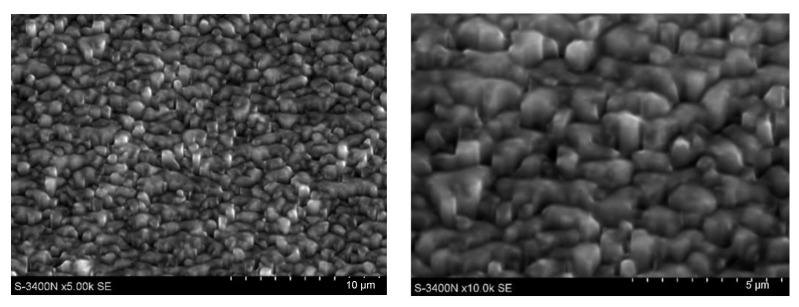
Microstructure of the WE43 alloy extruded using KoBo after compression at 400 °C and at a strain rate of 0.1 s^−1^ (ion etching).

Due to difficulties with the preparation of metallographic specimens for KoBo extruded and compressed samples, ion etching by means of a FIB device was applied, using a gallium beam of different strength. The analysis carried out with the use of the electron microscopy revealed ultrafine recrystallized grains (Figure 10) with a size smaller than that of those compressed after conventional extrusion under the same process conditions.

Table 5 shows the results obtained in a static tensile test for samples after conventional extrusion and KoBo extrusion at 300 °C and 350 °C and at a strain rate of 0.0001 m·s^−1^. The tensile test conducted at temperatures of 300 °C and 350 °C for the WE43 alloy after deformation using the KoBo method showed better plastic properties. At 300 °C, the obtained elongation amounted to 85%. At 350 °C, superplastic flow and elongation values of 250% were observed. 

The WE 43 alloy extruded using the KoBo method, subjected to tension at ambient temperature, showed slightly higher strength and plastic properties than those of the alloy extruded using the conventional method. This mainly resulted from the higher microstructure refinement (smaller grain size). Most likely, due to the accumulation of large plastic strain caused by the cyclic, oscillatory motion of the extrusion die, greater homogenization of the alloy occurred.

Specimens extruded using the KoBo method, subjected to tension at elevated temperatures, exhibited much higher elongation than that of the conventionally deformed specimens due to easier sliding across grain boundaries of the material characterized by a much smaller grain size. Under suitable temperature conditions and a tensile speed of 350 °C/0.0001 s^−1^, the alloy became superplastic. This is also indicated by the values of the parameter m determining the strain rate sensitivity shown in Figure 8. Similarly, the lower strengthening of the alloy samples extruded in a combined manner is due to the higher propensity for dynamic recrystallization in the fine-grained material.

## 4. Discussion

The effect of the applied deformation methods on changes in the microstructure, mechanical properties, and yield characteristics of the WE43 alloy was analyzed. Two extrusion methods were used: the classic one—concurrent extrusion, and concurrent extrusion with a reversible die (KoBo). The fineness of the grains in relation to the initial state was due to the dynamic recrystallization, which occurred during deformation. A finer grain size was found in the microstructure of the WE43 alloy extruded using the KoBo method than that observed when using the classical concurrent method of extrusion. The WE43 alloy extruded in the classical way is characterized by a much greater variation in grain size compared to the microstructure of the alloy after extrusion using the KoBo method, where the microstructure is more homogeneous. Examination of the microstructure indicated that the dynamic recrystallization process for the WE43 alloy deformed at a temperature of 300 °C and higher. At 250 °C, only elongated grains were observed, with samples failing before reaching the set strain ε = 1. Full (complete) recrystallization of the alloy was observed after compression at 350 °C and at a strain rate of 0.01 s^−1^.

For the other process parameters used, the extent of recrystallization depended on the strain rate. For both extrusion methods studied, increasing the temperature and lowering the strain rate favored a reduction in yield stress. A particularly large reduction in yield stress with a decreasing strain rate was observed after KoBo extrusion. Such behavior is characteristic of fine-grained materials, which show high sensitivity to the strain rate.

This is probably due to the tendency of the fine-grained material to undergo dynamic recrystallization and to more quickly reach the critical value to initiate the microstructure reconstruction process, hence the lower strengthening. The occurring recrystallization process favorably affected the formability of the WE43 alloy after deformation with the KoBo method. Differences in the Z parameter values for both analyzed variants resulted from the considerably lower activation energy Q for the alloy subject to deformation in the combined manner (Q-253.9 kJ/mol) than that for the alloy that was subject to a conventional deformation process (Q-389.2 kJ/mol) (Table 3). The lower value of the activation energy indicates a much higher susceptibility of the WE43 alloy, after combined extrusion, to hot forming at a lower temperature. At 350 °C, after extrusion with the KoBo method, a superplastic flow effect was observed after a static tensile test, with an elongation value of 250% (Table 5). The test results indicate a much higher plastic formability of the WE43 alloy extruded using the complex KoBo method at elevated temperatures, i.e., 350 °C, than that observed when using concurrent extrusion.

Available literature data indicate the use of SPD methods to shape the microstructure and properties of the WE43 alloy. The authors of paper] [19] used equal channel angular pressing, while in paper [20] the authors used multi-axial forging (MAF). The results obtained in the indicated publications confirm the achievement of the superplasticity effect for the studied WE43 alloy. The results obtained by these authors became the basis for undertaking this research with the aim of applying another SPD method, the KoBo method.

## 5. Conclusions

1. The fineness of the grains in the microstructure of the WE43 alloy extruded using the KoBo method was found to be higher than that observed when using the classic extrusion method.
2. Examination of the microstructure indicated a dynamic recrystallization process for the WE43 alloy deformed at 300 °C and above that temperature. For the other process parameters used, the degree of recrystallization depended on the strain rate. Full recrystallization was observed after compression at 350 °C and a strain rate of 0.01 s^−1^.
3. A significant reduction in yield stress with a decreasing compression rate was observed after extrusion with the KoBo method.
4. Activation energy for the WE43 alloy was determined to be 253.9 kJ/mol after KoBo extrusion and 389.2 kJ/mol after concurrent extrusion.
5. The effect of superplastic flow of the WE43 alloy after applying the extrusion process with the KoBo method was determined, and a 250% elongation value was achieved.

## Figures and Tables

**Table 1:** Chemical composition of the WE43 alloy (mass %).

Chemical Composition (Mass %)									
Alloy WE43	Y	HRE + Nd	Nd	Zr	Zn	Si	Cu	Ag	Fe
	4%	3%	2.2%	0.54%	0.03%	0.01%	0.01%	0.01%	0.002%

**Table 2:** Results of quantitative characterization of the WE43 alloy after applying the KoBo method.

WE43 Alloy	Average Surface Area Ā (µm2)	Coefficient of Variation S(A) (%)	Average Equivalent Diameter of Grains (µm)	Shape Factor
after casting	99	150.3	11.2	0.73
after hot extrusion, rods with diameter of 8 mm	68	214.3	9.3	0.68
after cold extrusion with KoBo method, rods with diameter of 8 mm	55	160.4	8.4	0.67
after cold extrusion with KoBo method, rods with diameter of 6 mm	1	120.3	1.1	0.53

**Table 3:** The determined values of flow stress σ_pmax_ and the corresponding strain ε_p_ after an axial-symmetric compression test in the temperature range of 250–350 °C at a strain rate of 0.01–1 s^−1^.

Temperature	Strain Rate	Classic Extrusion		Extrusion with KoBo Method	
[°C]	$\dot{\varepsilon }$ [s^−1^]	σ_pmax_	ε_p_	σ_pmax_	ε_p_
250	0.01	325	0.40	300	0.6
300	0.01	226	0.42	138	0.06
	0.1	278	0.57	225	0.47
350	0.01	133	0.15	40	0.12
	0.1	172	0.36	117	0.06
	1	208	0.41	153	0.2
400	0.1	105	0.09	52	0.08

**Table 4:** Calculated constants in Equations (2) and (6) for the investigated WE43 alloy.

Alloy—WE43	After Classic Extrusion	After Extrusion with KoBo Method
Q (kJ/mol)	398.20 kJ/mol	253.94 kJ/mol
n(-)	10.19	3.06
α (MPa^−1^)	0.00479	0.01161
C (s^−1^)	1.187 × 10^32^	7.558 × 10^19^
U(-)	0.00074	0.00000
w(-)	0.079	0.242

**Table 5:** Results of tensile testing of the investigated alloy at increased RT, 300 °C, and 350 °C after KoBo method. Temperature (°C).

	Classic Extrusion			Extrusion KoBo Method		
	R_p0,2_ [MPa]	R_m_ [MPa]	A [%]	R_p0,2_ [MPa]	R_m_ [MPa]	A [%]
RT	210	242	18	220	265	24
300	120	160	45	96	108	85
350	60	76	80	32	40	250

## Data Availability

Data are contained within the article.
